# Methyl-β-cyclodextrin, an actin depolymerizer augments the antiproliferative potential of microtubule-targeting agents

**DOI:** 10.1038/s41598-019-43947-4

**Published:** 2019-05-21

**Authors:** Nikita Mundhara, Abhijit Majumder, Dulal Panda

**Affiliations:** 1 Department of Biosciences and Bioengineering, Indian Institute of Technology Bombay, Mumbai, 400076 India; 2 Department of Chemical Engineering, Indian Institute of Technology Bombay, Mumbai, 400076 India

**Keywords:** Cancer, Biophysics

## Abstract

Methyl-β-cyclodextrin (MCD), an established pharmacological excipient, depolymerizes the actin cytoskeleton. In this work, we investigated the effect of MCD-mediated actin depolymerization on various cellular phenotypes including traction force, cell stiffness, focal adhesions, and intracellular drug accumulation. In addition to a reduction in the contractile cellular traction, MCD acutely inhibits the maturation of focal adhesions. Alteration of contractile forces and focal adhesions affects the trypsin-mediated detachment kinetics of cells. Moreover, MCD-mediated actin depolymerization increases the intracellular accumulation of microtubule-targeting agents (MTAs) by ~50% with respect to the untreated cells. As MCD treatment enhances the intracellular concentration of drugs, we hypothesized that the MCD-sensitized cancer cells could be effectively killed by low doses of MTAs. Our results in cervical, breast, hepatocellular, prostate cancer and multidrug-resistant breast cancer cells confirmed the above hypothesis. Further, the combined use of MCD and MTAs synergistically inhibits the proliferation of tumor cells. These results indicate the potential use of MCD in combination with MTAs for cancer chemotherapy and suggest that targeting both actin and microtubules simultaneously may be useful for cancer therapy. Importantly, the results provide significant insight into the crosstalk between actin and microtubules in regulating the traction force and dynamics of cell deadhesion.

## Introduction

Cyclodextrins are extensively used as adjuvants to make drugs more soluble, stable and bioavailable^1,2^. They are biocompatible, water-soluble, stable macro-molecules and are extensively used for drug delivery both as nano-carriers and solubilizer^3–5^. Some of its derivatives are also approved by FDA for human usage and do not trigger an immune response in human^6^. Methyl-beta-cyclodextrin (MCD), one of such derivatives, is extensively used to increase the permeability of cells^7^, and thereby increase the uptake of small molecules such as glucose^8^ and nano-particles^9^. MCD has also been reported to depolymerize the actin cytoskeleton in the cells^10,11^. Actin plays vital roles in several cellular processes such as cell migration, cell division, cytokinesis and also maintenance of cell shape and size^12^. The depolymerization of actin not only affects these functions but also increases plasma membrane permeability in various types of cells^13^. Increase in permeability by actin depolymerization allows higher uptake of small molecules, electrolytes, and drugs^14^. However, the effect of MCD on the actin-dependent physiological functions of a cell has not been studied in details.

In this study, we first sought to investigate the effect of MCD on the cytoskeleton of cells and also examined the physical consequences of the perturbation of the actin network in the presence of MCD. Cell physiological parameters like traction force, cell stiffness, deadhesion kinetics as well as the maturation of focal adhesions were studied in MCD treated and untreated cells. In addition, we performed an in-depth analysis of the combined effect of actin depolymerization by MCD and microtubule perturbation by MTAs on traction force and deadhesion kinetics of the cells. Interestingly, we found that the depolymerization of actin overrides the effect of microtubule perturbation in controlling the cellular traction.

Further, MCD treatment increased the intracellular accumulation of microtubule-targeting agents (MTAs) as reported with other cytotoxic drugs^15,16^. Prior treatment with MCD strongly increased the efficacy of vinblastine and taxol in breast, liver, cervical cancer and multi-drug resistant breast cancer cells. The combined use of MCD with MTAs provides a new avenue to enhance the antiproliferative potential of the MTAs. It also indicates a possibility that the perturbation of actin network may be combined with the perturbation of microtubules for successful cancer chemotherapy.

## Results

### MCD depolymerized the actin cytoskeleton but did not perturb the microtubules

**Figure 1:**
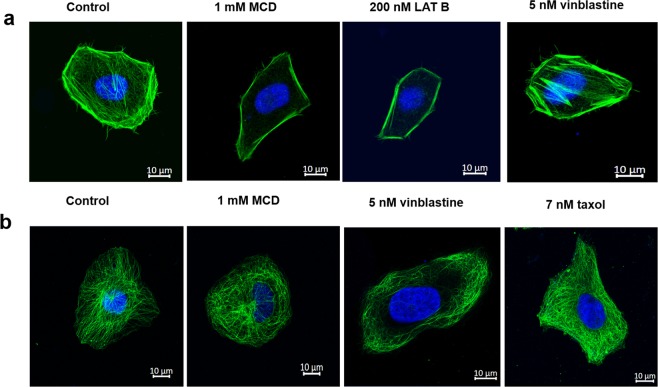
Effects of MCD on the actin and microtubule network in HeLa cells. HeLa cells were incubated in the absence or the presence of 1 mM MCD for 4 h. (**a**) Actin was stained by Phalloidin 488 and shown in green and the nucleus stained with Hoechst is shown in blue. 200 nM LAT B for 4 h was used as a positive control. (**b**) Microtubules were stained with β-tubulin antibody and represented in green while the nucleus is shown in blue. Vinblastine and taxol were used as positive controls (N = 3). The fluorescence intensity of both actin and microtubules were quantified using Image J (n = 100). MCD (<0.01) and LATB (<0.01) treatment significantly depolymerized the actin network.

HeLa cells were incubated with 1 mM MCD for 4 h and the F-actin was stained with phalloidin. MCD treatment reduced the fluorescence intensity of phalloidin-stained actin filaments by 49 ± 3% (p < 0.01) indicating that it depolymerized the actin network (Fig. 1a). Latrunculin B (LAT B), a pharmacological inhibitor of actin polymerization^17^, reduced the fluorescence intensity of the actin network by 37 ± 6% (p < 0.01) while vinblastine treatment showed no noticeable change in the actin network as compared to the control HeLa cells. There was no discernible change in the microtubules of HeLa cells upon 1 mM MCD treatment while vinblastine depolymerized microtubules and taxol enhanced microtubule assembly (p < 0.01) (Fig. 1b & Supplementary Table S1).

### MCD treatment altered focal adhesion expression, cell stiffness, traction force and deadhesion rates in HeLa cells

**Figure 2:**
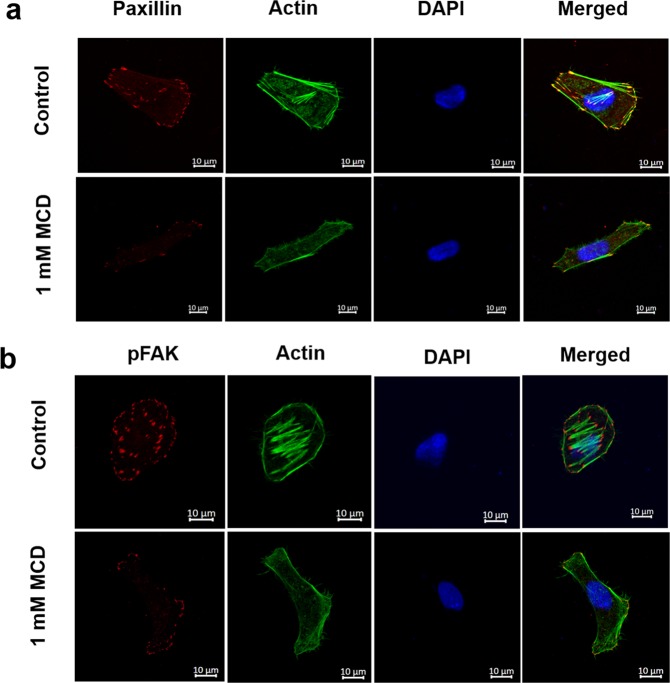
MCD treatment reduced the expression of initial and mature focal adhesions. HeLa cells were incubated in the absence or the presence of 1 mM MCD for 4 h. (**a**) Co-staining of paxillin (red), actin (green), nucleus (blue) was done with an anti-paxillin antibody, phalloidin, and Hoechst respectively. (**b**) Co-staining of pFAK (red), actin (green), nucleus (blue). The focal adhesion area was quantified by Image J. (n = 100). The reduction in the size of both paxillin (focal adhesion) and pFAK (focal adhesion kinase) area in MCD treated cells with respect to their untreated controls is significant (p < 0.01).

**Figure 3:**
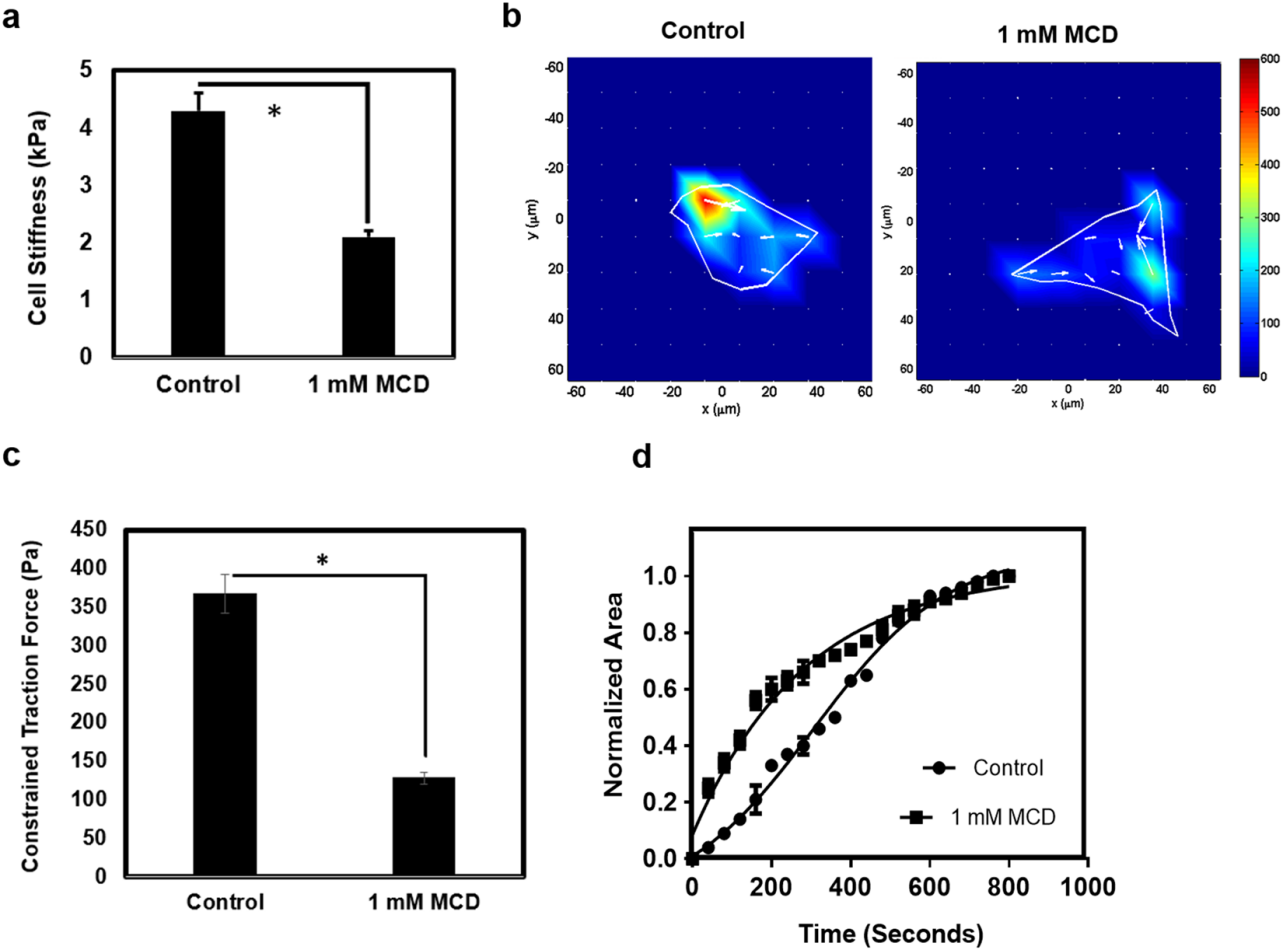
MCD treated cells show altered cell stiffness, traction force, and deadhesion rates. HeLa cells were incubated in the absence or the presence of 1 mM MCD for 4 h. (**a**) Bar graph showing cell stiffness (k Pa) of MCD treated and untreated HeLa cells as measured by atomic force microscopy (n = 50) and (*p < 0.01). (**b**) Constrained traction force heat map representing traction force of control and MCD treated HeLa cells as measured by TFM. (**c**) Bar graph showing constrained traction force (Pa) of MCD treated and untreated HeLa cells as measured by traction force microscopy (n = 30) and (*p < 0.01). (**d**) Normalized area v/s time plot of control and MCD treated HeLa cells upon trypsin treatment (n = 30). Data are averages of three independent experiments, and error bars represent the SD.

MCD-treatment depolymerized the actin network and therefore, we examined the effect of MCD on various actin dependent physiological parameters like focal adhesions, cell stiffness, traction force and deadhesion kinetics in HeLa cells. The focal adhesion assembly of HeLa cells was examined by immunostaining with antibodies against initial focal adhesion protein paxillin and matured focal adhesion protein phosphorylated focal adhesion kinase protein (pFAK). The expression of paxillin and pFAK was analyzed by determining the focal adhesion area. The paxillin focal adhesion area was reduced by 56% from 0.53 ± 0.04 to 0.23 ± 0.01 µm^2^ (p < 0.01) and pFAK focal adhesion kinase area reduced by 66% from 0.57 ± 0.07 to 0.23 ± 0.04 µm^2^ (p < 0.01) when the cells treated with 1 mM MCD for 4 h (Fig. 2a,b). An analysis using Atomic force microscopy (AFM) indicated that MCD treatment reduced the stiffness of HeLa cells by 50% from 4.3 ± 0.3 to 2.1 ± 0.2 kPa (p < 0.01) (Fig. 3a). The MCD-treated cells manifest a reduction in the traction force by 65% from 367 ± 15 to 128 ± 11 Pa (p < 0.01) as measured by traction force microscopy (TFM) (Fig. 3b,c). The rate of cell deadhesion from the substratum is a function of cell adhesion and traction force^18^. As MCD affected both cellular adhesion and traction (Figs 2a,b and 3b,c), it was interesting to study their combined effects on the deadhesion kinetics of HeLa cells. The deadhesion kinetics was studied by trypsin deadhesion assay that provided two-time constants τ_1_ and τ_2_^18^. τ_1_ indicates the time required for an initial reduction in cell area by 50% whereas τ_2_ indicates the time required for a further 50% to 75% reduction in the cell area. τ_1_ depends on both cell adhesion and cellular traction whereas τ_2_ depends only on the traction force^18^. We found a significant reduction in τ_1_ and a modest increase in τ_2_ in MCD – treated HeLa cells with respect to the controls (p < 0.01) (Fig. 3d & Supplementary Table S2).

### MCD treatment increased the intracellular accumulation of MTAs in HeLa cells

The depolymerization of the actin network has been suggested to increase the permeability of the plasma membrane^14,19^. Therefore, we wanted to know the effect of MCD mediated actin depolymerization on intracellular accumulation of small molecule inhibitors of tubulin. For this purpose, three inhibitors of microtubules namely BODIPY vinblastine (boron-dipyrromethene vinblastine)^20,21^ crocin^22^ and curcumin^23^ were chosen. MCD treatment increased the intracellular accumulation of BODIPY vinblastine, crocin and curcumin by 53 ± 3, 50 ± 3 and 50 ± 4%, respectively. Further, to check that MCD mediated actin depolymerization was the cause for the increase in the plasma membrane permeability, LAT B, a known inhibitor of actin polymerization, was used as a positive control. LAT B treatment also enhanced the intracellular accumulation of BODIPY vinblastine and curcumin in HeLa cells by 35 ± 4 and 30 ± 6%, respectively.

### MCD treatment strongly enhanced the antiproliferative effects of vinblastine, taxol, and crocin in several cancer cells including the multi-drug resistant ones

**Figure 4:**
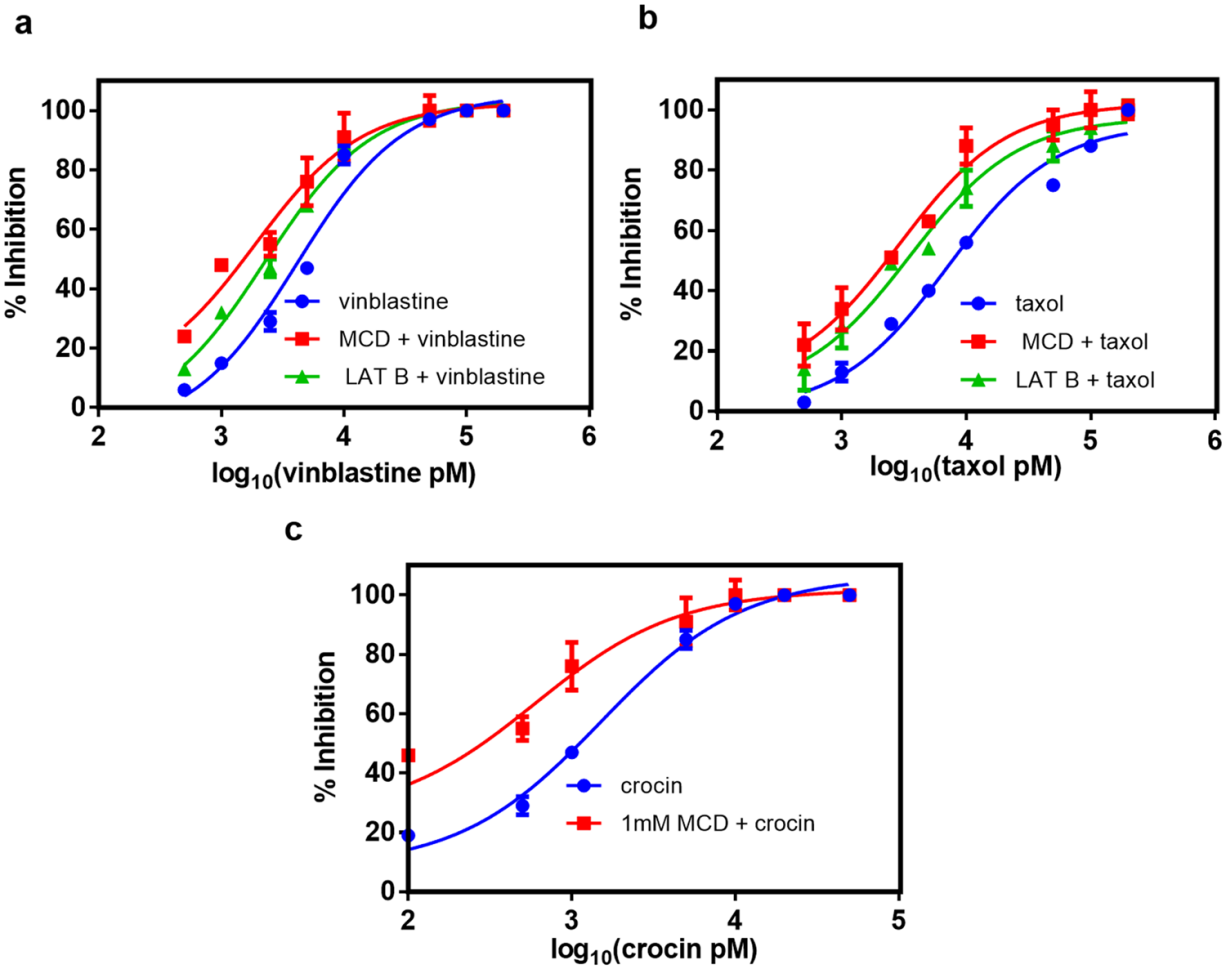
MCD augmented the anti-proliferative activity of vinblastine, taxol, and crocin in HeLa cells. HeLa cells were treated with either 1 mM MCD or 200 nM LAT B for 4 h (positive control) and then replaced with either fresh media alone or fresh media containing an increasing concentration of either vinblastine, taxol, crocin for 24 h. The inhibition of cell proliferation was calculated by the sulforhodamine B assay. (**a**) Inhibition of cell proliferation by vinblastine in control and MCD/LATB treated cells (p < 0.01). (**b**) By taxol in control and MCD/LAT B treated cells (p < 0.01). (**c**) By crocin in control and MCD treated cells (p < 0.01). Data are averages of three independent experiments, and error bars represent the SD.

As MCD treatment increased the intracellular accumulation of MTAs in HeLa cells, we investigated the effect of MCD in combination with clinically used MTAs like vinblastine and taxol against HeLa cells. To study the combined effects of MCD and MTAs, HeLa cells were initially treated with 1 mM MCD for 4 h. Then, the media with MCD was removed by washing the cells with the fresh media containing no MCD. The pre-treatment of cells with 1 mM MCD had no effect on the proliferation of HeLa cells up to 24 h. However, higher concentrations of MCD inhibited the proliferation of HeLa cells (Supplementary Fig. S1a). Subsequently, the cells were cultured in fresh media without and with different concentrations of either vinblastine, crocin or taxol. We found that the antiproliferative activity of vinblastine, crocin and taxol against HeLa cells is potentiated by pre-treatment with MCD. Vinblastine, taxol and crocin inhibited the proliferation of MCD-treated HeLa cells with an IC_50_ value of 1.9 ± 0.2 nM, 2.7 ± 0.3 nM and 0.6 ± 0.2 µM, respectively, which are significantly (p < 0.01) lower than vinblastine, taxol and crocin alone 4.2 ± 0.1 nM, 7 ± 0.3 nM and 1.5 ± 0.1 µM, respectively (Fig. 4a–c). In this experiment, LAT B was also used as a positive control. HeLa cells were pre-treated with 200 nM LAT B for 4 h. Upon pre-treatment with LAT B, vinblastine and taxol inhibited HeLa cell proliferation with an IC_50_ value of 2.3 ± 0.2 (p < 0.01) and 3.5 ± 0.2 (p < 0.01) nM, respectively indicating that the pre-treatment with LAT B could sensitize the cells towards the anti-microtubule agents. Further, we examined whether the pre-treatment with MCD could increase the antiproliferative effects of vinblastine and taxol in several other cancer cell lines. The antiproliferative effects of vinblastine and taxol were strongly enhanced in the liver (Huh 7) (Supplementary Fig S2a,b), breast (MCF-7) (Supplementary Fig. S2c,d), prostate (PC3) (Supplementary Fig. S2e,f) cancer cells when the cells were pre-treated with MCD. The augmented anti-proliferative effects of vinblastine and taxol on MCD sensitized several cancer cells encouraged us to investigate the potential of this drug combination on multi-drug resistant mouse breast mammary carcinoma (EMT/AR1) cells. The IC_50_ value of vinblastine was reduced from 3.7 ± 0.6 µM to 1 ± 0.05 µM (p < 0.01) and that of taxol from 1.2 ± 0.03 µM to 0.5 ± 0.02 µM (p < 0.01) indicating that MCD can sensitize drug-resistant cells towards MTAs (Supplementary Fig S2g,h). Supplementary Table S3 summarizes the IC_50_ values of vinblastine and taxol on control and MCD treated several cancer cell types.

### The anti-mitotic effect of vinblastine on cell cycle arrest was enhanced by MCD treatment

**Figure 5:**
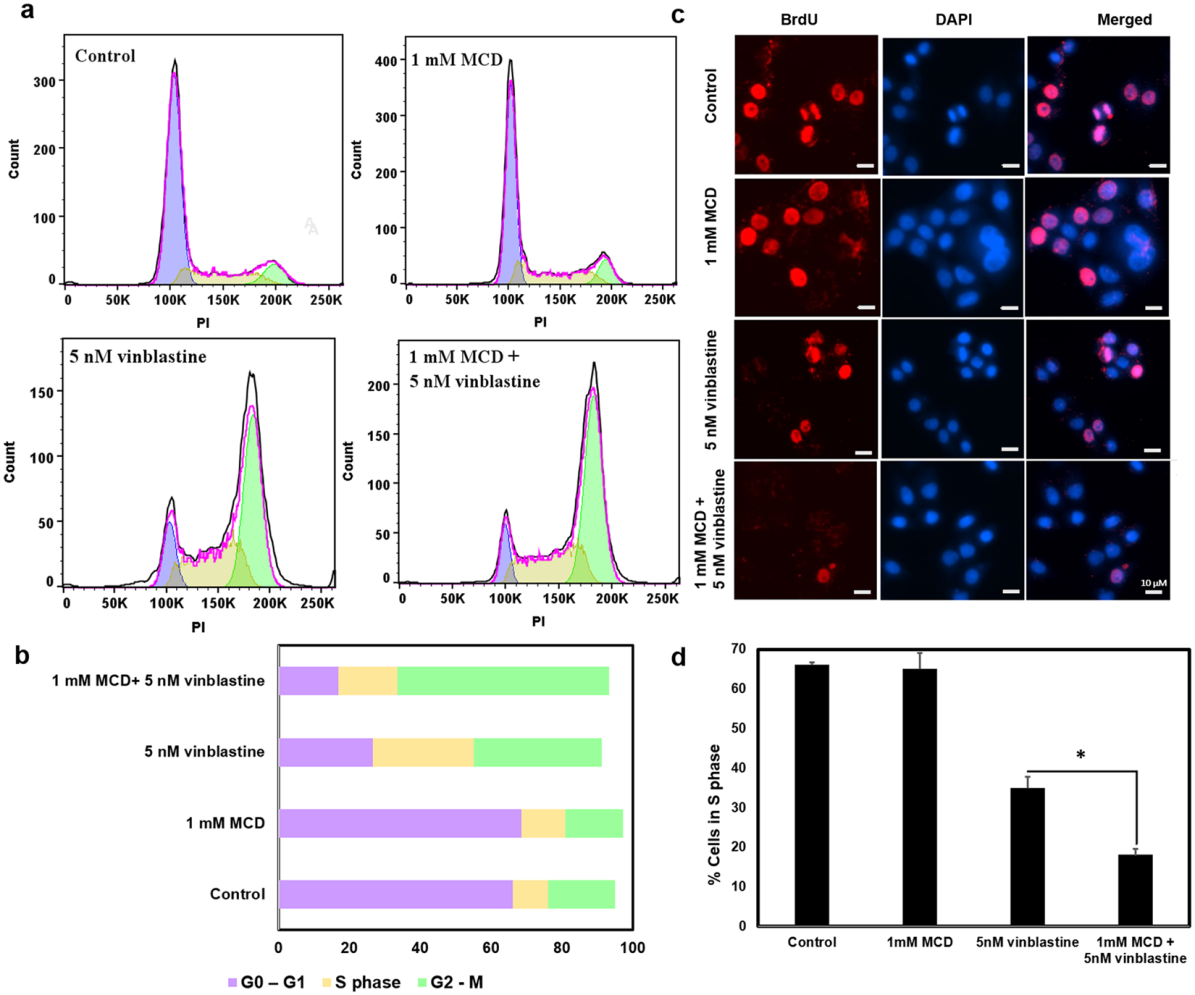
MCD potentiated the effect of vinblastine on the G2/M block. HeLa cells were treated with MCD and then replaced with either fresh media alone or fresh media containing 5 nM vinblastine for 24 h. Samples were stained with PI and subsequently were subjected to cell cycle analysis using a flow cytometer. (N = 2) (**a**) Flow cytogram showing the distribution of cells in different phases of the cell cycle using FLOW JO software. (**b**) Percentage bar graph showing the distribution of cells in different phases of the cell cycle. (**c**) MCD treated and untreated HeLa cells administered with 5 nM vinblastine for 8 h and then subjected to 4 h BrdU treatment followed by staining with anti- BrdU antibody and Hoechst. Representative images of BrdU Assay (pink) cells in S phase while (blue) cells except for S phase (n = 30). (**d**) A bar graph representing the percentage of cells in S phase. Data are averages of three independent experiments, and error bars represent the SD. The reduction in the percentage of cells in S phase after 8 h vinblastine treatment in MCD pre-treated cells with respect to their untreated controls is significant (*p < 0.01).

The antiproliferative effect of vinblastine and taxol was found to increase upon pre-treatment of cells with 1 mM MCD for 4 h (Fig. 4). As vinblastine is known to block the HeLa cells at mitosis^24^, it was important to investigate the effect of vinblastine on the cell cycle of MCD-treated HeLa cells. We found that prior treatment with MCD increased vinblastine (5 nM) mediated G2/M block from 37 ± 2% to 60 ± 4% (p < 0.01) (Fig. 5a,b). As the cells are blocked in the G2-M phase, we expected a significant reduction in the number of cells in S phase. BrdU (5-Bromo-2′-deoxyuridine) assay, which estimates the percentage of cells in the S phase of the cell cycle^25^, confirmed the prediction. The percentage of cells in the S phase was reduced from 66 ± 0.7% in control to 35 ± 2.9% after 8 h of vinblastine treatment. When the cells were incubated with MCD for 4 h prior to the addition of vinblastine, the percentage of cells in the S phase was further reduced to be 18 ± 1.6% (p < 0.01) (Fig. 5c,d).

### MCD treatment augmented the effect of vinblastine, taxol, crocin on both interphase and mitotic microtubules

**Figure 6:**
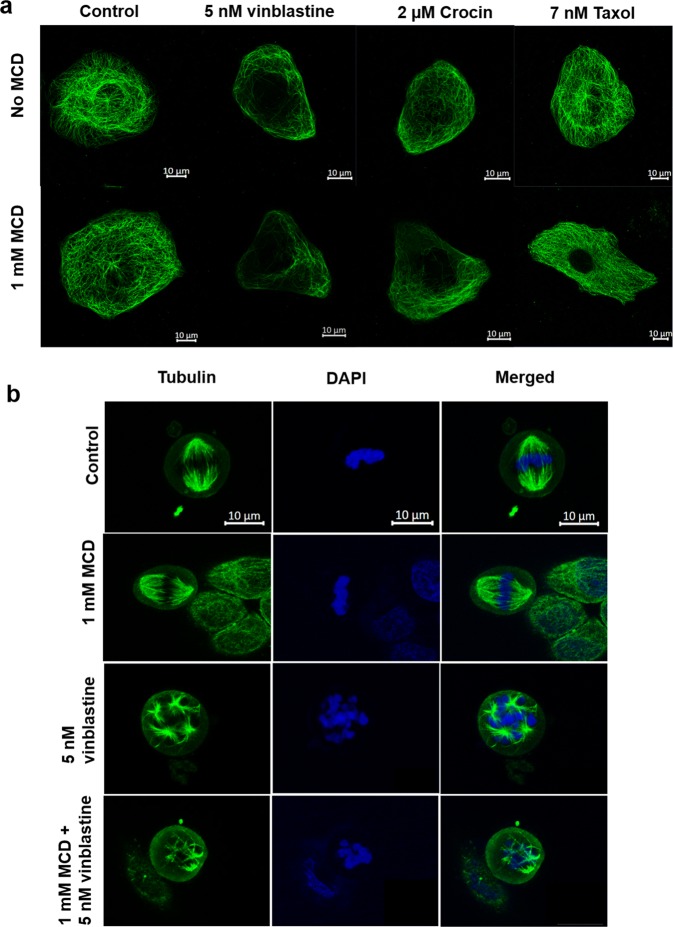
MCD augmented the effect of vinblastine, taxol, and crocin on both interphase and mitotic microtubules. HeLa cells were incubated in the absence or the presence of 1 mM MCD for 4 h. Then, the media was replaced with either fresh media or fresh media containing either 5 nM vinblastine or 7 nM taxol or 2 µM crocin for 8 h. (**a**) Interphase Microtubules (MT) are shown in green. The fluorescence intensity of microtubules was calculated by Image J (n = 100). MCD pre-treated and untreated cells upon vinblastine (p < 0.01), taxol (p < 0.01) and crocin (p < 0.05) treatment show significant difference. (**b**) Mitotic microtubules are shown in green and the nucleus stained with Hoechst is shown in blue (N = 3).

MCD treatment had no discernible effect on the microtubules in HeLa cells (Fig. 1b). However, prior treatment with 1 mM MCD for 4 h was found to enhance the effects of vinblastine, crocin, and taxol on microtubules of both interphase (Fig. 6a) and mitotic HeLa cells (Fig. 6b). The fluorescence intensities are summarized in Supplementary Table S1. For example, while 5 nM vinblastine treatment alone reduced the fluorescence intensity of microtubules by 43% of the control, pre-treatment with MCD reduced it further to 62%. Similarly, the effects of crocin and taxol on microtubules were found to be potentiated upon MCD treatment.

### The interplay of actin and microtubule assembly on the traction force and adhesion dynamics of HeLa cells

**Figure 7:**
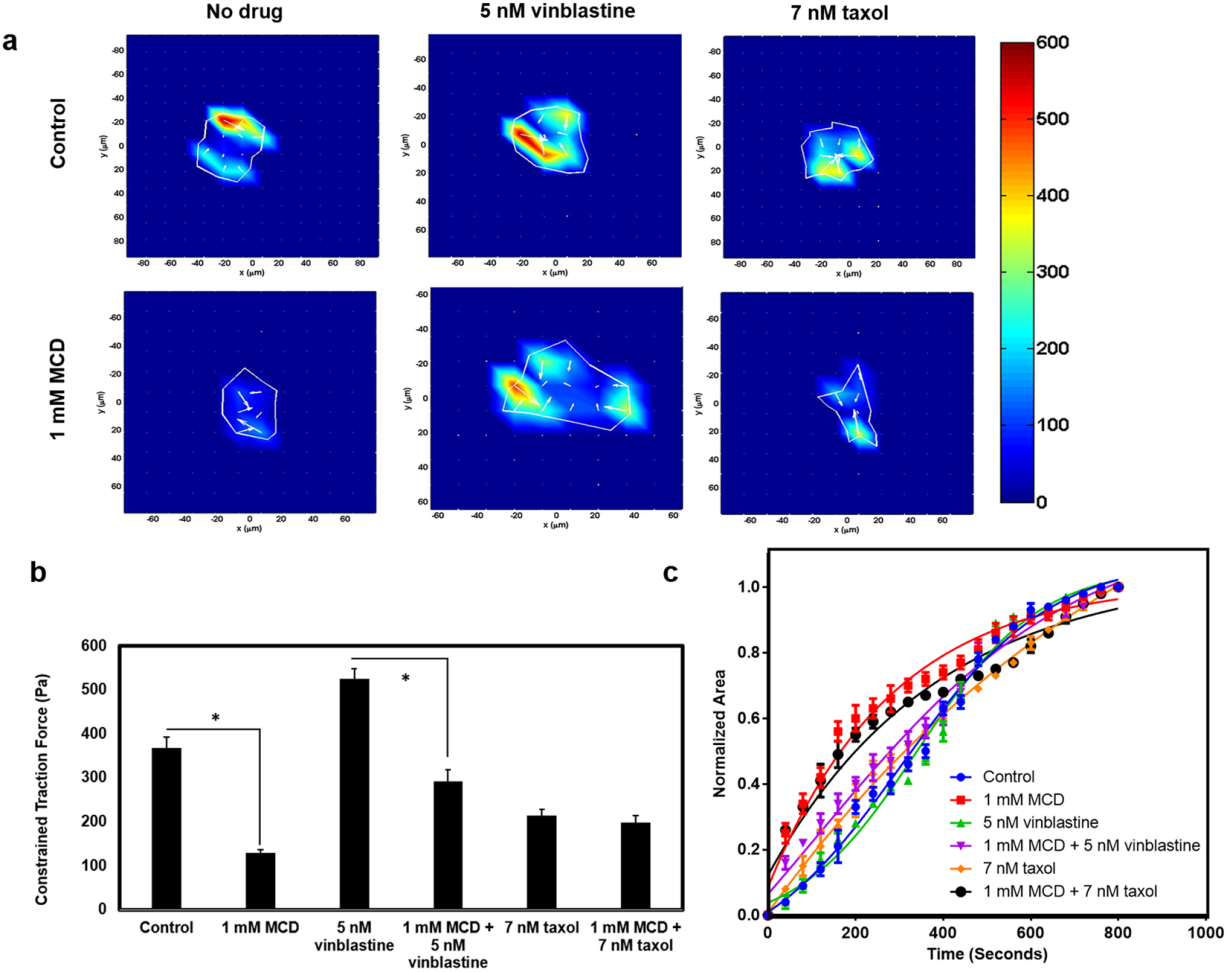
The combined effects of MCD with either vinblastine or taxol on the traction force and cell deadhesion kinetics. Cells were treated with 1 mM MCD for 4 h and then incubated with indicated drug concentration for 8 h. (**a**) Traction force heat maps of control and various drug concentrations are indicated as obtained by TFM (N = 3). (**b**) Bar graph representation of constrained traction force where data are averages of 30 cells each and error bars represent the SD. Control and 1 mM MCD (p < 0.01), Control and 5 nM vinblastine (p < 0.01), Control and 7 nM taxol (p < 0.01), 5 nM vinblastine and MCD + 5 nM vinblastine in (p < 0.01), 7 nM taxol and 1 mM MCD + 7 nM taxol (p = 0.33). (**c**) Trypsin deadhesion assay was done by adding trypsin after drug incubation and images were captured at an interval of 25 seconds (N = 3). Normalized area v/s time plot of 30 cells each of indicated MCD and MTAs combination (n = 30) and error bars represent the SD.

We wanted to comprehend the combined effects of MCD and MTA on cellular contractility and cell deadhesion kinetics, by TFM and trypsin deadhesion assay, respectively. As shown earlier (Fig. 3b,c), MCD treatment reduced the traction force from 367 ± 25 Pa to 128 ± 8 with respect to the control. However, 5 nM vinblastine treatment increased the traction force to 524 ± 24 Pa. A prior 4 h MCD treatment followed by vinblastine showed somewhat intermediate traction force as 292 ± 25 Pa. Similarly, at 7 nm taxol treatment reduced the traction force to 214 ± 13 Pa and a combination with MCD is 198 ± 15 Pa (Fig. 7a,b). The effects of vinblastine and taxol on the detachment kinetics of the cell deadhesion were analyzed by determining the τ_1_ and τ_2_ values (Supplementary Table S2) as described earlier. Neither vinblastine nor taxol treatment significantly altered the τ_1_ values of HeLa cells. However, the τ_2_ values for vinblastine and taxol-treated cells indicate faster and slower deadhesion, respectively with respect to the control. MCD nullified the effect of vinblastine resulting in intermediate τ_1_ and τ_2_ values whereas MCD and taxol acted in synergy to increase both τ_1_ and τ_2_ values. (Fig. 7c).

### MCD and vinblastine exert a synergistic anti-proliferative effect on HeLa cells

**Figure 8:**
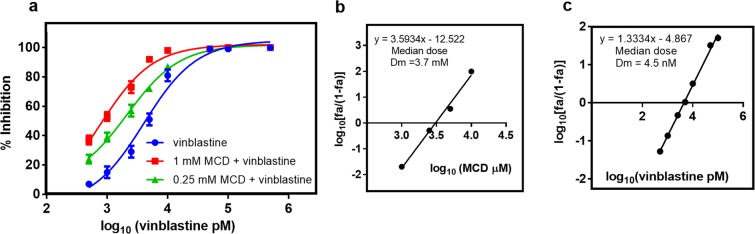
MCD and vinblastine synergistically inhibited HeLa cell proliferation. HeLa cells were treated with and without 1 mM and 0.25 mM MCD in the presence of different concentrations of vinblastine for 24 h. (**a**) The inhibition of cell proliferation in the presence of different concentrations of vinblastine and 1 mM MCD + different concentrations of vinblastine (p < 0.01), 0.25 mM MCD + different concentrations of vinblastine (p < 0.01). (**b**) The inhibition of cell proliferation for MCD and (**c**) vinblastine are shown. Median doses are calculated from the plots. Data are averages of three independent experiments, and error bars represent the SD.

The MCD-sensitized cancer cells exhibited a significantly higher inhibition of cell proliferation against vinblastine and taxol as compared to the control cells. Therefore, we evaluated the inhibitory effect of vinblastine on HeLa cell proliferation when used in combination with either 0.25 or 1 mM MCD for 24 h. The IC_50_ value of vinblastine was determined to be 0.7 ± 0.1 and 2.1 ± 0.2 nM in the presence of 1 and 0.25 mM MCD, respectively (Fig. 8a). The median dose of vinblastine and MCD was determined to be 4.5 nM and 3.7 mM, respectively (Fig. 8b,c). Vinblastine in combination with 1 and 0.25 mM MCD was found to synergistically inhibit the proliferation of HeLa cells with a combination index of 0.43 and 0.55, respectively^26,27^.

## Discussion

Consistent with the earlier reports^10,11^, MCD treatment was found to disassemble the actin network. In this work, we showed that MCD treated HeLa cells manifest a decrease in the expression of both early and mature focal adhesions proteins such as paxillin and pFAK, respectively. Using traction force microscopy, MCD-treatment was found to reduce both the traction force and cell stiffness. MCD treatment also enhanced the permeability of the cell membrane and thereby allows higher uptake of small molecules such as BODIPY vinblastine, crocin, and curcumin. LAT B and MCD enhanced the intracellular accumulation of BODIPY vinblastine by 35 and 53%, respectively indicating that the depolymerization of actin network may be the cause for the enhanced intracellular drug accumulation. However, the difference in the level of drug uptake also suggests that an additional mechanism may exist for MCD to further enhance the membrane permeability.

The traction force of a cell is a resultant of actomyosin generated contractile force and counteracting microtubules^28^. Perturbations to any of these cytoskeletal elements are expected to change the resultant traction force^29,30^. This work provides evidence of variations in the traction force and deadhesion kinetics of the cell when both actin and microtubules were perturbed simultaneously. We observed that the depolymerization of actin overrides the effect of the microtubule perturbation on the traction force. Vinblastine treatment depolymerized microtubules and increased the traction force. However, a 4 h prior MCD treatment, which disassembled the actin, almost nullified the vinblastine-induced increase in the traction force. This is because the actomyosin complex is responsible for the generation of cellular traction and microtubules acts as a counteracting stabilizer to this force. The de-adhesion kinetics of a cell upon trypsin treatment is a combined function of cellular traction and focal adhesions. While traction facilitates the deadhesion process, strong focal attachments decelerate it^18,31^. τ_1_ is dependent on the combined effect of focal adhesions and traction while τ_2_ estimates the contractile force alone^18^. Upon MCD treatment, the cells are de-adhering faster (τ_1_) as compared to controls due to the effect of weaker focal adhesions while the τ_2_ values increased due to the reduction in the traction force. However, vinblastine and taxol-treated cells did not show a significant change in τ_1_ values with respect to the control. The unaltered τ_1_ values suggest that focal adhesions are not altered by the administration of a low dosage of tubulin inhibitors though literature suggests that higher doses of the tubulin inhibitors modulate the expression of focal adhesion proteins^32,33^. The effect of altered traction force upon vinblastine and taxol treatment can be seen from the varying τ_2_ values with respect to control. The increased and decreased traction force in case of vinblastine and taxol treatment, respectively, had an opposite effect on the τ_2_ values. Further, simultaneous perturbation of the actin and microtubule networks by MCD and MTAs manifested intermediate τ_1_ values. This can be due to the combined effect of reduced focal adhesion by MCD and unaltered focal adhesion upon treatment with MTAs. MCD’s action in terms of cellular traction is opposite to vinblastine and in combination, they enhance the τ_2_ values as compared to both control and vinblastine alone. This phenomenon further confirms that actomyosin generated contractile force supersedes the counter stabilizing action of microtubules. Further, taxol and MCD together worked synergistically to enhance the τ_2_ values significantly because both acted compositely to reduce the traction force. This study illustrates the interplay between actin and microtubules on traction force and deadhesion kinetics of the cells and provides a deeper insight towards understanding the force dynamics played by the cytoskeletal elements of the cell.

MCD has been used to enhance the bioavailability and solubility of several drug candidates^1,4^. In this work, we show that MCD sensitized multiple cancer cells such as cervical, breast, liver, and prostate cancer showed a potentiated response to MTAs such as vinblastine, taxol, and crocin^22,34^. Further, to show that this augmented cell inhibition response was due to the depolymerizing effect of MCD on the actin network, LAT B a well-known inhibitor of actin polymerization was used as a positive control. A very low dosage of LAT B enhanced the antiproliferative efficacy of vinblastine and taxol. This confirms that MCD mediated actin depolymerization synergistically acts with MTAs to increase their efficacy in cell inhibition. Additionally, we show that the multi-drug resistant breast cancer cell line EMT/AR1 also show promising response when pre-treated with MCD. Actin targeted anti-proliferative drugs are highly toxic and therefore are not used for cancer chemotherapy^35,36^. As reported earlier, we have also found that MCD at higher doses leads to cell death^37,38^. In this work, we used the concentration of MCD which didn’t perturb the cell proliferation rather increases the intracellular drug accumulation of MTAs by actin depolymerization. Additionally, we found that simultaneous use of low concentration of MCD with vinblastine synergistically inhibits the proliferation of cancer cells. This study indicates that the perturbation of actin network by MCD along with microtubule depolymerization by MTAs provides an effective drug administration strategy to cancer patients who are struggling with drug resistance.

The present study indicates that MCD-induced actin depolymerization increases plasma membrane permeability, which allows the passage of more MTAs inside the cells and thereby increases their physiological concentrations. This sort of combination enables us to understand the crosstalk between microtubules and actin filaments and their inter-dependent roles in several physical parameters such as stiffness, adhesion and traction force of a cell. The results also suggest that devising a method to manipulate both actin and microtubules can be effective in devising new strategies to cancer therapeutic regimen.

## Methods

### Materials

Taxol, vinblastine, crocin, curcumin, methyl beta-cyclodextrin (MCD), mouse monoclonal anti-β tubulin, mouse monoclonal anti-paxillin IgG, mouse monoclonal anti-pFAK IgG, Alexa 488 flour phalloidin IgG, Alexa flour 564 rabbit anti-mouse IgG, FITC (fluorescein isothiocyanate) conjugated anti-mouse IgG, Alexa flour 568 goat anti-mouse, fetal bovine serum (FBS), bovine serum albumin (BSA), Propidium Iodide (PI), and Hoechst 33258 were purchased from Sigma (St. Louis, MO, USA). BODIPY vinblastine was purchased from Invitrogen. Taxol, vinblastine, crocin, and curcumin were dissolved in 100% Dimethyl sulfoxide while MCD was dissolved in Milli qH_2_O. (Solubility 50 mg ml^−1^). Other reagents were purchased from Genetix (India), Sigma-Aldrich, HiMedia (Mumbai, India), and Merck Millipore.

### Cell culture conditions

Human cervical carcinoma (HeLa), human breast adenocarcinoma (MCF-7), and hepatocarcinoma (huh7), prostatic small cell carcinoma (PC3) were purchased from cell repository of National Centre for Cell Science, Pune, India. A multidrug resistant derivative of mouse mammary carcinoma EMT6/ AR1 was purchased from Sigma (St. Louis, MO, USA). After the procurement, all the cell lines were passaged 3–4 times, collected and frozen. HeLa, MCF-7, Huh7, PC3 and EMT6 / AR1 cells were grown in Dulbecco Modified Essential Medium (DMEM) (HiMedia, India) containing fetal bovine serum (10%), 2.2 gl^−1^ sodium bicarbonate, and antibacterial and antimycotic solution (1%) containing streptomycin, amphotericin B, and penicillin in a carbon dioxide incubator (Sanyo, Tokyo, Japan) at 37 °C as described earlier^39,40^.

### Approach for MCD and drug integrated treatment

Cells were incubated with either 1 mM MCD or 200 nM LAT B for 4 h. Then, the media containing either MCD or LAT B was carefully removed and the cells were washed with fresh medium without MCD and LAT B. The cells were grown in the absence and presence of different concentrations of vinblastine, taxol, crocin, and curcumin until and unless specified otherwise.

### Determination of cell proliferation

Cells (1 × 10^5^ cells ml^−1^) were seeded in a 96 well cell culture plate. After cell attachment, the medium is then restored and cells were incubated with a fresh medium comprising of 1 mM MCD or 200 nM LAT B for 4 h. Consequently, it was replaced by either the vehicle (0.1% DMSO) or varying drug concentrations. The cells were allowed to proliferate for one life cycle and cell proliferation was estimated by widely used Sulphorhodamine B assay^41^ by monitoring absorbance at 520 nm using a SpectraMax M2^e^ Microplate reader as described earlier^42^. The concentration of a compound required for 50% inhibition of cell proliferation is defined as its IC_50_ value. The IC_50_ value was determined by nonlinear regression fitting of the concentration-dependent proliferation inhibitory data^43^.

### Cell cycle analysis by flow cytometry

Cells were seeded in T 25 flasks and allowed to reach 70% confluency and were treated with 1 mM MCD for 4 h. Then, MCD was removed and the cells were incubated with 5 nM vinblastine for 24 h. Subsequently, cells were processed and analyzed by a flow cytometer (FACS Caliber, Becton Dickinson) as described earlier^39,44^. Briefly described, cells were fixed in 70% (v/v) ethanol, incubated with 10 μg ml^−1^ RNase A and 50 μg ml^−1^ PI for 30 min. The DNA content of the cells was determined by a flow cytometer. The cell cycle distribution was analyzed using the FlowJo software.

### BrdU assay

Cells (1 × 10^5^ cells ml^−1^) were seeded on glass coverslips for 16 h and then, the cells were treated with 5 nM vinblastine for 8 h. In the case of MCD treatment, cells were first treated with 1 mM MCD for 4 h and then, the cells treated with 5 nM vinblastine for 8 h. After the drug treatment, BrdU reagent (Roche Diagnostics) was added in 1:1000 (v/v) ratio in media and incubated for 4 h at 37 °C with 5% CO_2_. Cells were processed for immunofluorescence^45^. Cells were incubated with anti-BrdU antibody (DSHB, University of Iowa) at 1:200 dilution in 1.5% BSA overnight at 4 °C followed by incubation with secondary antibody goat anti-mouse Alexa flour 568 at 1: 400 dilution for 3 h. Cell nuclei were stained with Hoechst 33258 at 1:10000 dilution. Immunofluorescence images were acquired and the percentage of BrdU positive cells were counted manually using Image J software.

### Immunofluorescence microscopy for microtubule, actin and focal adhesion staining

Cells were seeded at a density of 0.5 × 10^5^ cells ml^−1^ on glass coverslips. After 16 h they were treated with 1 mM MCD for 4 h and consequently with the drug for 8 h. After the incubation, cells were processed for immunofluorescence staining using specific antibodies as described earlier^40,46^. Cells were incubated with anti-mouse β-tubulin (1:300) antibody, anti-mouse paxillin antibody (1: 200) and anti-mouse pFAK antibody (1:200) for early and mature focal adhesion protein, respectively overnight at 4 °C followed by labeling with FITC conjugated anti-mouse secondary IgG (1:400) for microtubules and Alexa Flour 564 rabbit anti-mouse for focal adhesions for 1 h at room temperature. Actin was stained using Alexa flour 488 Phalloidin. Hoechst 33258 (1:10000) was used to stain the nucleus. Images were captured using a laser scanning confocal microscope (Zeiss 780) and processed using Zeiss ZEN software. The size of focal adhesions and the fluorescence intensity of actin and microtubules were determined using ImageJ software. A total of 100 cells were scored in each case.

### Drug accumulation assay

Cells were seeded in T 25 flasks and allowed to reach 50% confluency. HeLa cells were treated with 1 mM MCD for 4 h. The media with MCD was then carefully removed and then cells were subsequently treated with a fresh media containing with either 5 nM BODIPY vinblastine for 6 h or 1 μM crocin and 5 μM curcumin for 24 h. Cells were trypsinized using (0.05%) trypsin (HiMedia, India) containing (0.02%) EDTA (Sigma – Aldrich) followed by two PBS wash containing soybean trypsin inhibitor (HiMedia, India). The cell pellet was suspended in fresh 1 ml PBS and the cells were counted by hemocytometer. An equal number of cells for each condition was lysed by incubating in lysis buffer (150 mM NaCl, 0.1% Triton X-100, 50 mM Tris-HCl pH 8.0) for 1 h on ice with 10 s vortex every 10 min. The cell lysate was centrifuged at 19000 rpm for 20 minutes at 4 °C. The BODIPY vinblastine fluorescence of the supernatant was monitored at 513 nm using 485 as the excitation wavelength. The absorbance of the supernatant comprising of the cytosolic extract was taken at 425 and 440 nm for crocin and curcumin, respectively. The intracellular drug concentration for crocin and curcumin was determined from the absorbance.

### Trypsin cell deadhesion assay

Cell deadhesion study was performed using time-lapse microscopy as described^18,47^. Cells were seeded (0.5 × 10^5^ cells ml^−1^) density on glass coverslips. Following drug treatment, they were observed under Live-cell Imaging microscopy (EVOS FL, Life technologies, Invitrogen). Trypsin (0.05%) was added and images were captured at an interval of 25 s until the cells are completely de-adhered. Cell area quantification was done using ImageJ software and time constants were calculated using the Boltzmann linear regression curve fit on GraphPad Prism software.

### Traction force microscopy

Traction force microscopy was done as described earlier^47,45^. A 5 kPa gel was made using 40% (w/v) acrylamide and 2% (w/v) bis-acrylamide stock, (1:1000) 10% Ammonium persulfate, 10% and (1:10000) Tetramethylethylenediamine (TEMED) on 22 × 22 mm^2^ coverslips. For making TFM gels, 25 µl of gel solution was mixed with red fluorescent beads of 1 µm (Fluka) in 1:50 to dilution with APS and TEMED solution and placed on the hydrophobic coverslip. The pre-casted solidified 5 kPa gel was placed on the top of it. After solidification, gels were treated with sulpho-sanpah (Pierce; Thermo Scientific) and incubated with collagen type I (50 μg ml^−1^) (Advanced Biomatrix) overnight. Later (0.5 × 10^5^ cells ml^−1^) cells were seeded on it. After 16 h they were treated with 1 mM MCD for 4 h and then with 5 nM vinblastine / 7 nM taxol for 8 h. Imaging was done using phase contrast microscopy (EVOS FL, Life technologies, Invitrogen). Both fluorescent bead and phase cell images were taken for live cells. Following that, cells were lysed using 1% Triton X-100 and images were taken for the fluorescent beads without disturbing the gels. The bead displacement and traction forces were calculated using the code from^48^ in MATLAB (MathWorks).

### Atomic force microscopy

Cells were seeded (0.5 × 10^5^ cells ml^−1^) density on glass coverslips. Following 1 mM MCD treatment for 4 h, atomic force measurements are taken with TR400PB silicon nitride pyramidal tip probe on MFP-3D (Asylum Research) under contact mode force. The spring constant of the cantilever was 0.02 Nm^−1^ and frequency is 10 kHz. The force curves were then fitted with the Hertz model provided within the software from Asylum research.

### Determination of combination index

HeLa cells were incubated with either 1 or 0.25 mM MCD and different concentrations of vinblastine for 24 h. The combination index (CI) was determined by the Chou and Talalay method^26^ to know whether the combination of MCD and vinblastine could inhibit HeLa cell proliferation synergistically or additively^27,34^. A CI of < 1 suggests a synergistic combination.

The following equation was used to determine the combination index^26^.

$${\rm{CI}}=\frac{({\rm{D}})1}{({\rm{Dx}})1}+\frac{({\rm{D}})2}{({\rm{Dx}})2}$$

(D)1 and (D)2 are the concentrations of MCD and vinblastine in a combination that produces a certain effect, and (Dx)1 and (Dx)2 are the concentrations of MCD and vinblastine, respectively individually producing the same effect. (Dx) was calculated from the following equation as given by Chou *et al*.^49^.

$$({\rm{Dx}})=\mathrm{Dm}\,{[\frac{{\rm{fa}}}{(1-{\rm{fa}})}]}^{1/{\rm{m}}}$$

Dm is the median dose, fa is the fraction affected while (1 − fa) is fraction unaffected. A logarithmic median dose plot is obtained from X-axis being a log of drug concentration whereas Y-axis is log(fa/(1 − fa). The y-intercept and slope (m) of the equation of this median dose plot is used to calculate the Dm value^49^.

$${\rm{Dm}}={10}^{-({\rm{y}}-{\rm{intercept}})/{\rm{m}}}$$

Thus, Dm obtained from above is used to calculate the Dx and thereafter the CI is calculated.

### Statistical analysis

Student’s t-test by (GraphPad Prism Statistical software) was used to determine the significance value. Error bars in figures indicate standard deviations.

## Supplementary information

Supplementary information
