# Supplementary material for: Methyl-β-cyclodextrin, an actin depolymerizer augments the antiproliferative potential of microtubule-targeting agents
Source: Sci Rep. 2019 May 21;9:7638. doi: 10.1038/s41598-019-43947-4 (PMC6529501; doi:10.1038/s41598-019-43947-4)
Supplement: Supplementary file 1 — Supplementary information [file 41598_2019_43947_MOESM1_ESM.pdf]

Supplementary Information

Methyl-β-cyclodextrin, an actin depolymerizer augments the antiproliferative potential of microtubule-targeting agents

Nikita Mundhara [1], Abhijit Majumder [2*], Dulal Panda [1*]

1. Department of Biosciences and Bioengineering, Indian Institute of Technology Bombay, Mumbai 400076, India. (abhijitm@iitb.ac.in)
2. Department of Chemical Engineering, Indian Institute of Technology Bombay, Mumbai 400076, India. (panda@iitb.ac.in)

Tables

Table S1: Effects of vinblastine, crocin and taxol on the microtubules in HeLa cells without and with MCD treatment.

	**Microtubule Intensity (a.u.)**	
	**No MCD**	**1 mM MCD**
**Control**	27.2 ± 1.2	26.3 ± 2
**5 nM vinblastine**	15.5 ± 2.2$^{\#}$	10.3 ± 1.8 *
**2 µM crocin**	20.4 ± 1.5$^{\#}$	14.9 ± 0.8 **
**7 nM taxol**	34.5 ± 3.5$^{\#}$	45.4 ± 3.8 *

Data were an average ± SD of three independent set of experiments (n =50 cells). ($p < 0.01$ = * / $^{\#}$) and ($p < 0.05$ = **), (*) represents comparison with their respective MCD untreated controls while ($^{\#}$) represents comparison with control (no MCD).

Table S2: Rate constant (τ_1 and τ_2) values of the cell deadhesion.

Conditions	τ_1 (s) *	τ_2 (s) #
**Control**	332 ± 7	131 ± 3
**1 mM MCD (4 h)**	192 ± 15 *	158 ± 4 #
**5 nM vinblastine (8 h)**	339 ± 5	115 ± 2#
**1 mM MCD (4 h) + 5 nM vinblastine (8 h)**	290 ± 14 *	148 ± 2#
**7 nM taxol (8 h)**	326 ± 8	156 ± 6#
**1 mM MCD (4 h) + 7 nM taxol (8 h)**	224 ± 5 *	197 ± 5#

Data were an average ± SD of three independent set of experiments. (n=30 cells). ($p < 0.01$ = */ #). (*) represents comparison with τ_1 control whereas (#) represents comparison with τ_2 controls.

Table S3: IC_{50} values of vinblastine and taxol in control and MCD treated cells.

Cell Line	Vinblastine (nM)	1mM MCD + vinblastine (nM)	Taxol (nM)	1 mM MCD + taxol (nM)
**MCF 7**	25.9 ± 1.1	9.6 ± 0.8 *	24.8 ± 0.7	10.3 ± 0.9 *
**Huh 7**	72.1 ± 5.9	25.6 ± 2.5 *	70.8 ± 2.6	20.7 ± 1*
**PC 3**	12.3 ± 0.2	9.8 ± 0.8 **	17.5 ± 1.7	11.7 ± 0.17 **

Data were an average ± SD of three independent set of experiments. ($p < 0.01$ = *) and ($p < 0.05$ = **)

Figure S1

a

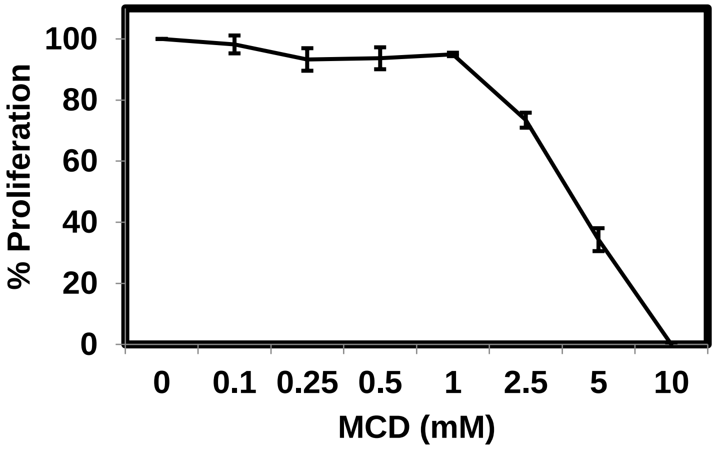

b

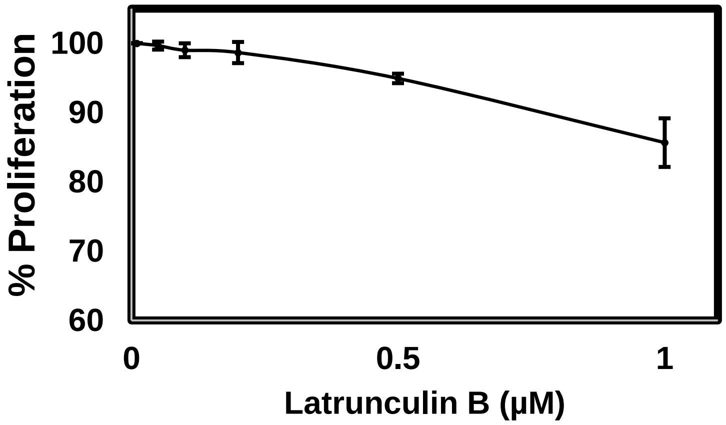

Supplementary Figure 1: MCD and LAT B inhibited the proliferation of HeLa cells at higher doses. HeLa cells were treated with different concentrations of MCD and LAT B for 24 h. The inhibition of cell proliferation was calculated by the sulforhodamine B assay. a) Line graph representing inhibition of cell proliferation upon increasing concentrations of MCD b) LAT B. Data are averages of three independent experiments, and error bars represent the SD.

Figure S2

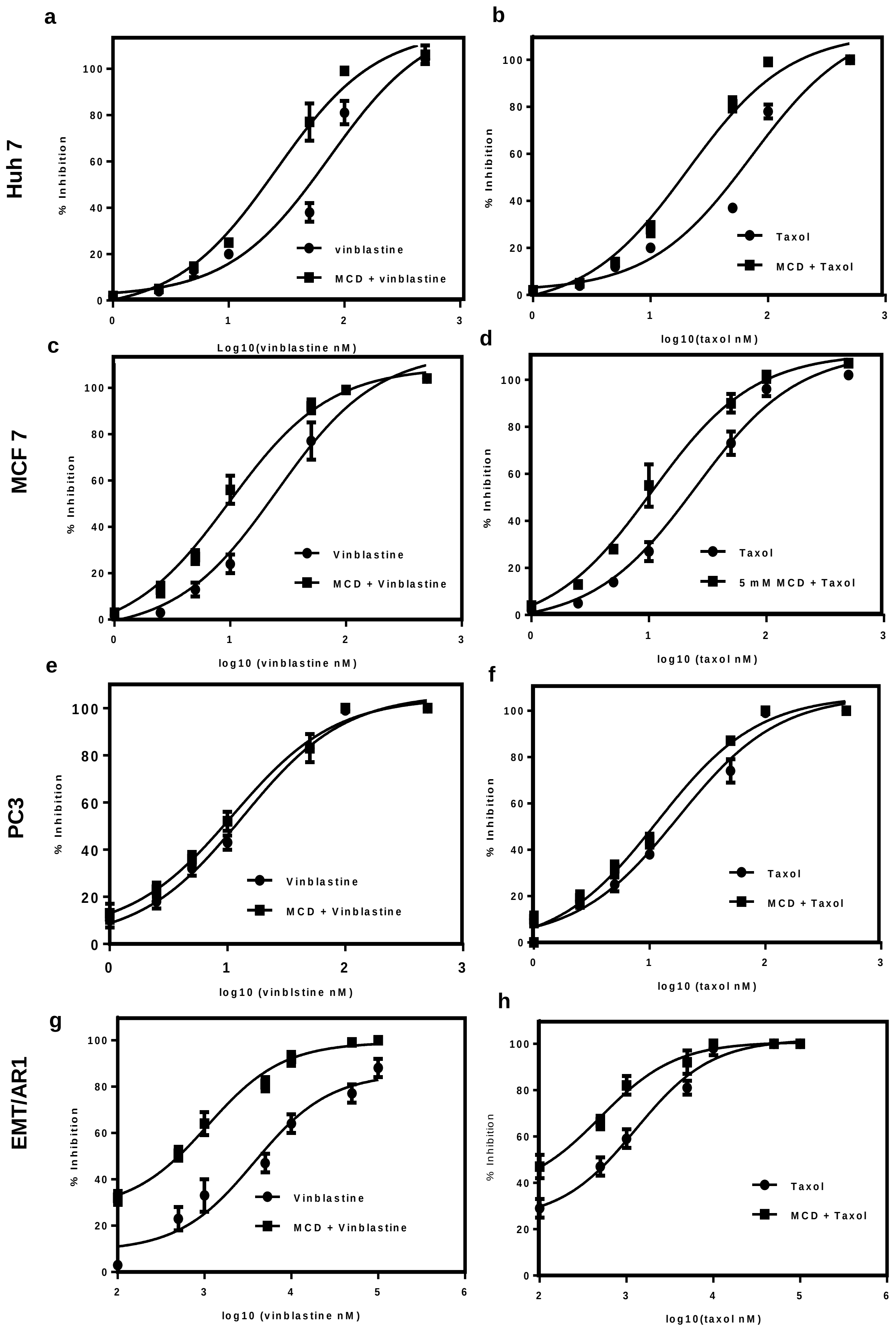
a
b
c
d
e
f
g
h
Huh 7
MCF 7
PC3
EMT/AR1
% Inhibition
Log10(vinblastine nM)
log10(taxol nM)
log10 (vinblastine nM)
log10 (taxol nM)
log10 (vinblstine nM)
vinblastine
MCD + vinblastine
Taxol
MCD + Taxol
Vinblastine
MCD + Vinblastine
5 mM MCD + Taxol

Supplementary Figure 2: MCD augmented the antiproliferative activity of vinblastine and taxol in several types of cancer cells. Cells were treated with 1 mM MCD for 4 h and then replaced with either fresh media alone or fresh media containing different concentrations of either vinblastine or taxol for 24 h. The inhibition of cell proliferation was calculated by the sulforhodamine B assay. a) Huh 7 cells - vinblastine ($p < 0.01$) b) Huh 7 cells - taxol ($p < 0.01$) c) MCF 7 cells - vinblastine ($p < 0.01$) d) MCF 7 cells - taxol ($p < 0.01$) e) PC3 cells - vinblastine ($p < 0.05$) f) PC3 cells - taxol ($p < 0.05$) g) EMT/AR1 cells - vinblastine ($p < 0.01$) f) EMT/AR1 cells - taxol ($p < 0.01$). Data are averages of three independent experiments, and error bars represent the SD.
